# Horsford’s Acid Phosphate

**Published:** 1888-02

**Authors:** 


HORSFORD'S ACID PHOSPHATE.

Nearly two years ago the editor of this Journal began to fear that a physical organization of which he was justly proud had been irretrievably injured by overtaxing its capacities, and by failure to observe proper periods of rest. He was a victim of indigestion, nervous depression and the horrors of insomnia. He was informed by competent medical authority that an entire vacation of some months would be necessary, but this seemed impracticable in view of the duties which devolved upon him. He was then recommended to commence the use of Horsford's Acid Phosphate, and to buy a horse, as the next best things. The former, that he might be certain of its quality, was obtained from the Rumford Chemical Works in Providence, R. I., and the latter was found nearer home. He has literally followed both, and cannot be certain which has given the most profit and pleasure, but knows that each has played an important part. The Acid Phosphate is certainly the cheapest, and, in this case, has proved best worth the money. After taking it for a time the old ambition and love for work returned, and the day's duties were no longer a dreary task. The victim of sleeplessness began to sleep well at night, and, indeed, when he found that ten hours no longer sufficed, began to think it time to discontinue the phosphate, or to give the day as well as the night up to slumber.

There are few vocations that make greater demands upon the nervous system than that of the operative dentist, and we believe that indigestion and wakefulness are more common among them than in almost any class of people. Very many such would find a great relief in the use of the Acid Phosphate. With sugar and water it makes a very pleasant drink, and for derangements of the secretive and nervous systems, it is especially useful. From personal use and knowledge we most heartily commend it to dentists who are sufferers from over-work and worry.

